# Phytochemicals from *Mangifera pajang* Kosterm and their biological activities

**DOI:** 10.1186/s12906-015-0594-7

**Published:** 2015-03-26

**Authors:** Sadikah Ahmad, Mohd Aspollah Sukari, Nurussaadah Ismail, Intan Safinar Ismail, Ahmad Bustamam Abdul, Mohd Fadzelly Abu Bakar, Nurolaini Kifli, Gwendoline C L Ee

**Affiliations:** Department of Chemistry, Faculty of Science, Universiti Putra Malaysia, Serdang, Selangor 43400 Malaysia; UPM-MAKNA Cancer Research Laboratory, Institute of Bioscience, Universiti Putra Malaysia, Serdang, Selangor 43400 Malaysia; Faculty of Science, Technology and Human Development, Universiti Tun Hussein Onn Malaysia, 86400 Parit Raja, Batu Pahat, Johor Malaysia; PAP Rashidah Sa’adatul Bolkiah Institute of Health Science, Universiti Brunei Darussalam, Jalan Tungku Link Gadong, BE1410 Brunei Darussalam

**Keywords:** *Mangifera pajang*, Bambangan, Phytochemicals, Cytotoxicity, DPPH, Antimicrobial

## Abstract

**Background:**

*Mangifera pajang* Kosterm is a plant species from the mango family (Anacardiaceae). The fruits are edible and have been reported to have high antioxidant content. However, the detailed phytochemical studies of the plant have not been reported previously. This study investigates the phytochemicals and biological activities of different parts of *Mangifera pajang*.

**Methods:**

The plant samples were extracted with solvents of different polarity to obtain the crude extracts. The isolated compounds were characterized using spectroscopic methods. The extracts and isolated compounds were subjected to cytotoxicity tests using human breast cancer (MCF-7), human cervical cancer (HeLa) and human colon cancer (HT-29) cells. The free radical scavenging activity test was conducted using the DPPH assay. Antimicrobial activity tests were carried out by using the disc diffusion method.

**Results:**

Phytochemical investigation on the kernel, stem bark and leaves of *Mangifera pajang* led to the isolation of methyl gallate (1), mixture of benzaldehyde (2) and benzyl alcohol (3), mangiferonic acid (4), 3*β*-hydroxy-cycloart-24-ene-26-oic acid (5), 3*β*,23-dihydroxy-cycloart-24-ene-26-oic acid (6), lupeol(7) lupenone(8), *β*-sitosterol(9), stigmasterol(10), *trans*-sobrerol(11) and quercitrin (12). Crude ethyl acetate and methanol extracts from the kernel indicated strong cytotoxic activity towards MCF-7 and HeLa cells with IC_50_ values of less than 10 μg/mL, while petroleum ether, chloroform and ethyl acetate extracts of the stem bark showed strong to moderate activity against MCF-7, HeLa and HT-29 cancer cell lines with IC_50_ values ranging from 5 to 30 μg/mL. As for the antimicrobial assays, only the ethyl acetate and methanol extracts from the kernel displayed some inhibition against the microbes in the antibacterial assays. The kernel extracts showed highest free radical scavenging activity with IC_50_ values of less than 10 μg/mL, while the ethyl acetate and methanol extracts of leaves displayed only weak activity in the DPPH assays.

**Conclusions:**

Phytochemical investigations on various parts of *Mangifera pajang* have identified terpenoids and a flavonol derivative as major constituents. Bioassay studies have indicated that the crude extracts and isolated compounds have potential as naturally-derived anticancer and antimicrobial agents, besides possess high free radical scavenging activity.

## Background

*Mangifera pajang* Kosterm is also known as ‘bambangan’; a plant species from the mango group which can be found in Borneo Island (Malaysia – Sabah and Sarawak, Brunei, and Indonesia – Kalimantan) [1]. Unlike commercial mangoes (*Mangifera indica*), the fruit of *Mangifera pajang* are rarely eaten as it is unpopular among the public in Peninsula Malaysia and it is relatively unknown as antioxidant source. The fruits are ovoid and light-brown coloured, while the peels are thick which constitute approximately 27% of the whole bambangan fruit. The bambangan fruit is also about three times larger than its more common counterpart, mango [2,3]. The pulp is fibrous, juicy, has a specific aromatic flavour and strong smell and can be eaten fresh while the peel is used for cooking curries [3]. In Sabah, the kernel and flesh are used to make ‘jerok bambangan’ among Kadazans, the young leaves are sold as vegetables in Sarawak, while the thick rind can be sun-dried and preserved to be used for preparation of ‘sambel’ [4]. Previous studies of the fruit parts (peel, pulp and kernel) have reported high antioxidant and cytotoxic activity towards cancer cell lines [5,6]. However, there are no reports on the isolation of chemical constituents from the kernel, leaves and stem bark of the plant. In continuation of our research on local underutilised medicinal plants, we wish to report herein on the phytochemical properties and biological activities of *Mangifera pajang.*

## Methods

### Plant collection

The plant was collected from Sabah, Malaysia and was identified by Dr Mohd Fadzelly Abu Bakar from Universiti Tun Hussein Onn Malaysia (UTHM). The voucher specimen was deposited at the herbarium, BORNEENSIS, Universiti Malaysia Sabah. The plant samples (kernel, leaves and stem bark) were air-dried and ground into a fine powder prior to being used.

### Extraction and isolation

The ground kernel (180 g), leaves (500 g) and stem bark (330 g) of *Mangifera pajang* were extracted successively, each three times with petroleum ether, chloroform, ethyl acetate and methanol respectively for three days, for each extract by cold maceration method. Small portions of each crude extract were put aside for bioassays and the rest were fractionated using the column chromatographic method. From the kernel of the plant, *β*-sitosterol (9) (7 mg) and a yellow oil containing mixture a of benzaldehyde (2) and benzyl alcohol (3) were obtained from petroleum ether (12.26 g) and chloroform extracts (1.30 g) while isolation work on the methanol extract (21.95 g) gave methyl gallate (1) (1.15 g). Various crude extracts; petroleum ether (18.08 g), chloroform (3.59 g), ethyl acetate (1.24 g) and methanol (5.04 g) extracts from stem bark of *Mangifera pajang* were fractionated using column chromatography which afforded mangiferonic acid (4) (3.10 g), 3*β*-hydroxy-cycloart-24-ene-26-oic acid (5) (2.27 g) and 3*β*,23-dihydroxy-cycloart-24-ene-26-oic acid (6) (10 mg), lupeol (7) (3.15 g), *β*-sitosterol (9) (9 mg), stigmasterol (10) (5 mg) and *trans*-sobrerol (11) (14 mg). In addition, fractionation of the leaves extract gave lupeol (7) (20 mg), lupenone (8) (16 mg), methyl gallate (1) (11 mg) and quercitrin (12) (1.29 g). The structures of the isolated compounds were elucidated using spectroscopic methods including infrared spectroscopy (IR), mass spectrometry (MS), and nuclear magnetic resonance spectroscopy (NMR).

**Methyl gallate (1)**: white solid, C_8_H_8_O_5_,m.p. 190–192°C (lit. m.p 188–189°C) [7]. IR (UATR, cm^−1^) ν_max_: 3347, 3100, 2850, 1686, 1441, 1466.EI-MS *m/z* (% intensity):184 (M^+^, 45). ^1^H and ^13^C NMR spectral data were in good agreement with the published data [7].

**Mixture of benzaldehyde (2) and benzyl alcohol (3)**: yellow oil, IR (UATR, cm^−1^) ν_max_: 3400, 1739, 1457. EI-MS *m/z* (% intensity) of benzaldehyde (C_7_H_6_O)**:** 106 (M^+^, 90). EI-MS *m/z* (% intensity) of benzyl alcohol (C_7_H_8_O):108 (M^+^, 77).

**Mangiferonic acid (4):** white solid, C_30_H_46_O_3_, m.p.185-188°C (lit. m.p. 188–192°C) [8]. IR (UATR, cm^−1^) ν_max_: 3306, 3067, 1681, 1642, 1454, 1376, 1106.EI-MS *m/z* (% intensity):454 (M^+^, 38).^1^H and ^13^C NMR spectral data were in good agreement with the published data [8].

**3*β*-Hydroxy-cycloart-24-ene-26-carboxylic acid (5)**: white solid, C_30_H_48_O_3_, m.p. 175–177°C (lit. m.p. 177–178°C) [8]. IR (UATR, cm^−1^) ν_max_: 3361, 2933, 2867, 1683, 1634, 1447, 1372, 1020. EI-MS *m/z*(% intensity):456 (M^+^, 9).^1^H and ^13^C NMR spectral data were in good agreement with the published data [8].

**3*β*,23-Dihydroxy-cycloart-24-ene-26-oic acid (6):** white needle-shaped crystals, C_30_H_48_O_4_, m.p. 276-278°C (lit. m.p 279–281°C) [9]. IR (UATR, cm^−1^) ν_max_: 3318, 3101, 2933, 2870, 1670, 1439, 1374, 1043. EI-MS *m/z*(% intensity):472 (M^+^, 6). ^1^H and ^13^C NMR spectral data were in good agreement with the published data [9].

**Lupeol (7):** white solid, C_30_H_50_O, m.p 199-210°C (lit. m.p 212-213°C) [10]. IR (UATR, cm^−1^) ν_max_: 3308, 2925, 2858, 1639, 1455, 1377, 1034. EI-MS *m/z*(% intensity):426 (M^+^, 7). ^1^H and ^13^C NMR spectral data were in good agreement with the published data [10].

**Lupenone (8):** Colourless needle-shaped crystals, C_30_H_48_O, m.p. 166–168°C (lit. m.p 168-170°C) [11]. IR (UATR, cm^−1^) ν_max_: 2938, 2861, 1702, 1643, 1451, 1380, 869. EI-MS *m/z*(% intensity):424 (M^+^, 43). ^1^H and ^13^C NMR spectral data were in good agreement with the published data [11].

***β*-Sitosterol (9):** white needle-shaped crystals, C_29_H_50_O, m.p. 133–135°C (lit. m.p 136-138°C) [12]. IR (UATR, cm^−1^) ν_max_: 3413, 2935, 2860, 1673, 1455, 1372, 1047. EI-MS *m/z*(% intensity):414 (M^+^, 49). ^1^H and ^13^C NMR spectral data were in good agreement with the published data [13].

**Stigmasterol (10):** colourless needle-shaped crystals, C_29_H_48_O, m.p. 168 to 170°C (lit. m.p 167 - 169°C) [14]. IR (UATR, cm^−1^)ν_max_: 3429, 2928, 2857, 1711, 1460, 1375, 1058. EI-MS *m/z* (% intensity):412 (M^+^, 10). ^1^H and ^13^C NMR spectral data were in good agreement with the published data [10].

***Trans*-sobrerol (11):** white needle-shaped crystals, C_10_H_18_O_2_, m.p. 130 to 132°C (lit m.p 130–133°C) [15]. IR (UATR, cm^−1^) ν_max_: 3413, 2935, 2865, 1673, 1455, 1372, 1047.EI-MS *m/z* (% intensity):170 (M^+^, 1).^1^H and ^13^C NMR spectral data were in good agreement with the published data [15].

**Quercitrin (12):** yellow solid, C_21_H_20_0_11_, m.p. 188–190°C (lit. m.p 179–182°C) [16]. IR (UATR, cm^−1^) ν_max_: 3429, 1653, 1599, 1498, 1062, 814. EI-MS *m/z* (% intensity):302 (M^+^- Rham). ^1^H and ^13^C NMR spectral data were in good agreement with the published data [16].

### Cytotoxic assays

Various crude extracts of *Mangifera pajang* were screened for cytotoxic activity against MCF-7 (human breast cancer), HeLa (human cervical cancer) and HT-29 (human colon cancer) cells according to the method described previously [17]. The stock solutions of 100 mg/mL were prepared by using dimethyl sulphoxide (DMSO) of concentrations ranging from 0.1 μg/mL – 30.0 μg/mL. Working solutions were made up by two fold dilution of the stock and 20 μL of each concentration was added to each well in triplicates. The control wells of the untreated population were treated with highest concentration DMSO as the negative control. After 3 days, the cell viability was determined by introducing 20 μL of MTT solution (5 mg/mL in PBS) to each well, followed by 4 hours of incubation. The blue formazan crystals that formed were dissolved in DMSO and the absorbance was read using the Elisa reader with wavelength of 570 nm and a reference wavelength of 630 nm. The cytotoxic activity was determined by the IC_50_ values, which were defined as the concentration of the test samples that resulted in a 50% reduction of absorbance or a measure of 50% concentration of tested samples that was required to inhibit the growth of cancer cells. Extracts and isolated compounds that indicated IC_50_ values < 10 μg/mL, are considered to have significant cytotoxic activity against that particular cell line.

### DPPH assay

The scavenging activity of the crude extracts and constituents were determined by using 1,1- diphenyl-2-pycrylhydrazyl (DPPH) according to a procedure described previously [18]. DPPH was used as the stable free radical agent while DMSO was used as the blank. The sample was dissolved in DMSO in the ratio of 1:1 (w/v) and diluted to achieve concentrations of 500, 250, 125, 62.50, 31.25, 15.63 and 7.81 μg/mL. The IC_50_ values were determined by plotting the percentage of inhibition against sample concentration, ranging from 500 to 7.81 μg/mL. The IC_50_ values are defined as the amount of antioxidants needed to decrease the initial concentration of DPPH by 50%.

### Antibacterial and antifungal assays

All crude extracts from *Mangifera pajang* (kernel, leaves and stem bark) were subjected to antibacterial and antifungal assays towards several targeted microbes including *Methicillin resistant staphylococcus aureus* (*MRSA*), *Pseudomonas aeruginosa*, *Salmonella choleraesuis* and *Bacillus subtilis* for antibacterial screening while *Candida albican*, *Aspergillus ochraceaus* and *Sacchoromyces cerevisiae* were used for antifungal screening. The assays were carried out using the disc diffusion method [17]. This involved placing paper discs of 6 mm in diameter that contained the samples onto a plate where the microbes were growing. Ampicillin (Gram-negative Bacteria) and Streptomycin (Gram-positive Bacteria) standards were used for the bacteria while nystatin was used as the positive control for the fungi. The plates were inverted and incubated at 30-37°C for 18–24 hours for bacteria and 24–48 hours for fungi or until sufficient growth had occurred. After incubation, each plate was examined. The diameters of the zones of complete inhibition were measured.

## Results and discussion

### Isolated constituents

Phytochemical investigations on the kernel, stem bark and leaves of *Mangifera pajang* have afforded several classes of constituents including aromatic esters, cycloartane and lupane triterpenes, a monoterpene, steroids and a flavonol glycoside.

**Figure 1:**
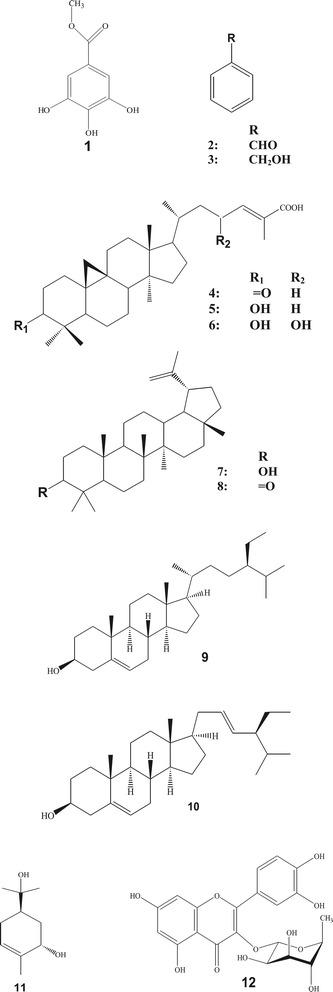
**Isolated constituents from** ***Mangifera pajang: methyl gallate*** **(1), mixture of benzaldehyde (2) and benzyl alcohol (3), mangiferonic acid (4), 3β-hydroxy-cycloart-24-ene-26-oic acid (5), 3β,23-dihydroxy-cycloart-24-ene-26-oic acid (6), lupeol (7), lupenone (8),** ***β*** **-sitosterol (9), stigmasterol (10),** ***trans*** **-sobrerol** **(11), quercitrin (12).**

- An aromatic ester; methyl gallate (1) and a mixture of benzaldehyde (2) and benzyl alcohol (3) together with *β*-sitosterol (9) have been isolated from the kernel. Cycloartane triterpenes; mangiferonic acid (4), 3*β*-hydroxy-cycloart-24-ene-26-oic acid (5) and 3*β*,23-dihydroxy-cycloart-24-ene-26-oic acid (6), lupeol (7), *β*-sitosterol (9), stigmasterol (10) and a monoterpene identified as *trans*-sobrerol (11) were obtained from the stem bark. Meanwhile lupane triterpenes; lupeol (7) and lupenone (8) together with methyl gallate (1) and a flavonol glycoside; quercitrin (12) have been isolated from the leaves. The structure of compounds shown in Figure 1 were elucidated using spectroscopic analysis (IR, EIMS and NMR) and also by comparison with reported data [7-16].
- Compound (1) was reported previously from ripe bambangan peel using HPLC-DAD and TSQ-ESI-MS analysis [19]. The compound was obtained from the methanol extracts of the kernel and leaves and which appeared as a white amorphous solid with m.p. of 190-192°C. Compound (2) and (3) were obtained as a yellow oily mixture from the petroleum ether and chloroform extracts of the kernel respectively. GCMS analysis of the oil suggested the presence of mixture of benzaldehyde (2) and benzyl alcohol (3). Molecular ion peaks at *m/z* 106 and 108 corresponded to benzaldehyde (2), C_7_H_6_O and benzyl alcohol (3), C_7_H_8_O, respectively.
- Compounds (4), (5), and (6) which belong to cycloartane type triterpenes were obtained from the non-polar extracts of the stem bark. These compounds were previously isolated from *Mangifera indica* [8,9] and this is the first reported isolation of the compounds from *Mangifera pajang*.
- Two lupane triterpenes, lupeol (7) and lupenone (8) were obtained from the extracts of the stem bark and leaves of *Mangifera pajang*. Lupeol (7) was obtained both from the stem bark and leaves while lupenone (8) was isolated only from the leaves. The EI-MS spectra of the compounds show molecular ion peaks at *m/z* 426 and 424 which corresponded to molecular formulae C_30_H_50_O (lupeol) and C_30_H_48_O (lupenone), respectively. Compound (9) and (10) which were identified as *β*-sitosterol and stigmasterol have also been isolated from the kernel and stem bark. The identities of the compounds were confirmed by comparing its physical and spectral data with the literature values [13,14].
- Compounds (11) and (12) were identified as *trans*-sobrerol and quercitrin, respectively. Compound (11) is a monoterpene while compound (12) is a flavonol glycoside and both compounds have never been isolated from the genus *Mangifera*. Compound (11) was isolated from chloroform extract of the stem bark and appeared as white needle crystals with a melting point of 130 - 132°C and the spectral data were in good agreement with the published data [15]. Meanwhile, compound (12) was obtained from the ethyl acetate and methanol extracts of the leaves and appeared as a yellow solid with a melting point of 188 - 190°C. The compound was identified by comparison of its spectral data with that available literature of the compound isolated previously from the leaves of *Myrsine seguinii* [16].

### Cytotoxicity of crude extracts and isolated compounds

All crude extracts from the kernel, stem bark and leaves were subjected to cytotoxic screening against MCF-7, HeLa and HT-29 cancer cells and the results are summarized in Table 1.

- Ethyl acetate and methanol extracts of the kernel showed strong cytotoxic activity towards MCF-7 and HeLa cell lines with IC_50_ values less than 10 μg/mL, and displayed strong to moderate activities towards the HT-29 cell line. In addition, petroleum ether and chloroform extracts of the stem bark also showed high cytotoxic activity towards MCF-7, HeLa and HT-29 cells with IC_50_ values of less than 15 μg/mL. In contrast, the ethyl acetate extract of the stem bark and the petroleum ether extract of the leaves displayed rather weak activity with IC_50_ values of more than 20 μg/mL. Similarly, most of the extracts from the leaves were not active with IC_50_ values being more than 30 μg/mL. Previous studies on the cytotoxic activity of *Mangifera pajang* have been reported on the ethanolic extract of its kernel towards MCF-7 (hormone-dependent breast cancer cells) and MDA-MB-231 (non-hormone dependent breast cancer cells) with IC_50_ values of 23 and 30.5 μg/mL, respectively [20]. For cytotoxic screening of the isolated compounds in this work, only six compounds with sufficient amounts were selected for the anticancer tests. Cytotoxic assay of the compounds against MCF-7 cell line (Table 1) indicated strong activity as shown by methyl gallate (1) with an IC_50_ value of 10.5 ± 0.29 μg/mL. Meanwhile, 3*β*-hydroxy-cycloart-24-ene-26-oic acid (5) displayed moderate cytotoxic activity towards MCF-7 with an IC_50_ value of 13.03 ± 0.81 μg/mL. In contrast, weak cytotoxic activities were shown by lupeol (7), lupenone (8) and quercitrin (12) with IC_50_ values of 25.02 ± 0.71 , 27.01 ± 0.34 and 25.04 ± 0.72 μg/mL respectively, while mangiferonic acid (4) was inactive with IC_50_ > 30 μg/mL.
- The results from cytotoxic tests of the chemical constituents of the plant against human cervical cancer (HeLa) cells (Table 1) indicated strong activity as was implicated by 3*β*-hydroxy-cycloart-24-ene-26-oic acid (5) with the IC_50_ value 6.27 ± 0.61 μg/mL. Apart from that, moderate activities were demonstrated by quercitrin (12), mangiferonic acid (4) and lupeol (7) with IC_50_ values of 11.93 ± 0.63, 16.51 ± 0.55 and 13.09 ± 0.80 μg/mL respectively. Other isolated constituents, methyl gallate (1) and lupenone (8) showed no activity with IC_50_ values > 30 μg/mL.
- Meanwhile, cytotoxic tests against human colon cancer (HT-29) cell (Table 1) indicated very strong cytotoxic activity shown by a flavonol glycoside, quercitrin (12) with IC_50_ values of 3.82 ± 0.91 μg/mL. In addition, methyl gallate (1) and mangiferonic acid (4) have also demonstrated moderate cytotoxic activity with IC_50_ values of 18.07 ± 0.37 and 18.03 ± 0.75 μg/mL, respectively. However, no cytotoxic activity were shown by 3*β*-hydroxy-cycloart-24-ene-26-oic (5), lupeol(7) and lupenone (8) where the IC_50_ values were more than 30 μg/mL.
- Some of the isolated compounds have been reported to show cytotoxic activities against several cancer cell lines. Methyl gallate (1) was shown to possess weak anticancer effects (IC_50_ value of more than 70 μg/mL) against human cervix adenocarcinoma cells (HeLa) and human fibroblast cells (L-132) [21]. However, a study by Lee et al. [22] demonstrated that methyl gallate (1) successfully enhanced antitumor effects through modulation of CD4+ CD25+ Treg cell functions, delaying tumour growth even though this compound was known to have low anticancer activity.
- Mangiferonic acid (4) reported by Li et al. [23] showed insignificant anticancer activity with IC_50_ values of around 100 μg/mL towards three murine cancer cell lines (colon 26-L5 carcinoma, B16-BL6 melanoma and Lewis lung carcinoma) and also towards three human cancer cell lines (lung A549 adecarcenoma, cervix HeLa adecarcinoma and HT-1080 fibrosarcoma).
- Meanwhile, anticancer activities of lupeol (7) as reviewed by Gallo and Sarachine, [24] found that lupeol (7) exhibited weak cytotoxicity in human melanoma SK-MEL-2, human lung carcinoma A549 and murine melanoma B16-F10 cells. Lupeol (7) also inhibited the proliferation of MDA-MB-231 human breast cancer cells and has been tested previously against many different numbers of cancer cells and showed range of activities towards the tested cells according to Gallo and Sarachine, [24]. These findings further corroborated with that reported in this work [25], where lupeol (7) and lupenone (8) exhibited weak cytotoxicity with IC_50_ values more than 30 μg/mL against human colorectal cancer (HT-29) and mammary breast cancer (MDA-MB) cell lines. However, lupeol (7) was slightly toxic against the normal cell line (3 T3) with an IC_50_ value of 38.92 μg/mL while lupenone (8) did not demonstrate any cytotoxic effect against the 3 T3 cell line with IC_50_ values of more than 100 μg/mL [26]. Meanwhile, quercitrin (12) was found to have a weak inhibitory effect on the human prostate cancer cell line PC-3 [26].

**Table 1:** **Cytotoxicity of** ***Mangifera pajang*** **isolates against various cancer cell lines**

**Mangifera pajang**	**Cytotoxic activities (IC**_**50**_ **μg/mL)**		
	**MCF-7**	**HeLa**	**HT-29**
Kernel (crude extracts)			
Petroleum ether	>30	>30	5.51 ± 0.98
Chloroform	15.60 ± 1.03	>30	23.06 ± 0.75
Ethyl acetate	3.99 ± 0.47	6.68 ± 0.46	14.40 ± 0.52
Methanol	4.12 ± 0.20	5.40 ± 0.53	4.94 ± 0.36
Stem bark (crude extracts)			
Petroleum ether	5.80 ± 0.23	11.8 ± 0.45	12.67 ± 0.82
Chloroform	11.26 ± 1.08	17.48 ± 0.62	12.11 ± 0.39
Ethyl acetate	>30	29.84 ± 1.03	15.01 ± 0.52
Methanol	>30	>30	>30
Leaves (crude extracts)			
Petroleum ether	>30	23.78 ± 0.73	23.19 ± 0.31
Chloroform	>30	>30	>30
Ethyl acetate	>30	>30	>30
Methanol	13.17 ± 0.49	>30	>30
Isolated compounds			
Methyl gallate (1)	10.53 ± 0.29	>30	18.07 ± 0.37
Mangiferonic acid (4)	>30	16.51 ± 0.55	18.03 ± 0.75
3β-hydroxy-cycloart-24-ene-26-oic acid (5)	13.03 ± 0.81	6.27 ± 0.61	>30
Lupeol (7)	25.02 ± 0.71	13.09 ± 0.80	>30
Lupenone (8)	27.01 ± 0.34	>30	>30
Quercitrin (12)	25.04 ± 0.72	11.93 ± 0.63	3.82 ± 0.91
*Standard	3.36 ± 0.02	4.93 ± 0.51	2.04 ± 0.03

*MCF-7: Tamoxifen, HeLa: Cisplatin, HT-29: 5-Fluorouracil.

IC_50_ > 30: Inactive, <10 μg/mL: highly active, 10 to 20 μg/mL: moderately active, 20 to 30 μg/mL: weakly active.

### Structure-activity relationships

The moderate to strong anticancer activities of the constituents of the plant have contributed to the overall cytotoxic properties of the plant extracts.

- Among the tested crude extracts, the methanol extract from kernel was the most potent with IC_50_ values less than 6 μg/mL for the three cancer cell lines. This may due to the presence of the cytotoxic methyl gallate (1) constituent. Methyl gallate (1) which is an ester (Figure 1), has a carbonyl and three hydroxyl groups attached to a benzene ring which probably synergistically enhanced the anticancer properties of the compound [21]. Meanwhile, mangiferonic acid (4) and 3β-hydroxy-cycloart-24-ene-26-oic acid (5) which were isolated from the hexane and ethyl acetate extracts of the stem bark of the plant, displayed various ranges of cytotoxicity against the three cancer cell lines. In the current study, 3β-hydroxy-cycloart-24-ene-26-oic acid (5) was more cytotoxic compared to mangiferonic acid (4). In contrast to the results described by Li et al. [23], the anticancer potency of the cycloartane skeleton was suggested to be depending on the substituent at C-3; in order of α-OH > C = O > β-OH [23].
- Quercitrin (12) obtained from extracts of leaves in polar solvents showed good anticancer activity with IC_50_ values around 10 μg/mL for HeLa and HT-29 cancer cell lines but simultaneously reduced the cytotoxicity of the crude extracts, suggesting the antagonistic effect of quercitrin (12). Quercitrin (12) is a glycoside of quercetin with a rhamnose group attached to the hydroxyl group at C-3. However, the glycoside substituent on the ring did not enhance the cytotoxicity of quercitrin (12) as its parent flavonol structure; quercetin significantly inhibited human prostate cancer cell line PC-3 cell proliferation, whereas quercitrin (12) itself did not have any antiproliferation effect on the cancer cells [26].
- In addition, lupeol (7) and lupenone (8) together with most of leaf extracts exhibited weak cytotoxicity towards all cancer cell lines. Lupeol (7) is slightly more cytotoxic compared to lupenone (8) due to the presence of a hydroxyl group at C-3.

### DPPH free radical scavenging activity

- Free radical scavenging activity was determined by DPPH assay (summarized in Table 2); where ethyl acetate and methanol extracts from the kernel showed high radical scavenging activity with IC_50_ values of less than 10 μg/mL, while polar (ethyl acetate and methanol) extracts of the leaves showed moderate to weak radical scavenging activity with IC_50_values 100 to 200 μg/mL. Previous studies have reported high scavenging activity of the fruit parts (peel, pulp and kernel) of *Mangifera pajang* [5], but antioxidant studies were not reported on its leaves and stem bark.
- Further DPPH screening assay on the isolated compounds indicated that methyl gallate (1) showed the highest radical scavenging activity with IC_50_ values of 6.24 ± 0.30 μg/mL, while mangiferonic acid (4), 3*β*-hydroxy-cycloart-24-ene-26-oic acid (5), lupeol (7) and lupenone (8) were not active with IC_50_ > 300 μg/mL. The strong scavenging activity of methyl gallate (1) was also reported previously [27], where they reported IC_50_ values of 2.8 μg/mL for methyl gallate in the DPPH free radical assay. However till date, there has been no previous report on free radical scavenging activity of mangiferonic acid (4) and 3*β*-hydroxy-cycloart-24-ene-26-oic acid (5).

**Table 2:** **DPPH free radical scavenging activity of isolates of** ***Mangifera pajang***

**Samples (** ***Mangifera pajang*** **)**	**Crude extracts/compounds**	**IC**_**50**_ **(μg/mL)**
**Kernel**	Petroleum ether	>300
	Chloroform	>300
	Ethyl acetate	7.28 ± 0.30
	Methanol	8.84 ± 1.04
**Stem bark**	Petroleum ether	>300
	Chloroform	>300
	Ethyl acetate	>300
	Methanol	>300
**Leaves**	Petroleum ether	>300
	Chloroform	>300
	Ethyl acetate	104.05 ± 1.02
	Methanol	186.26 ± 0.99
**Isolated compounds**	(1)	6.24 ± 0.3
	(4), (5), (7), (8)	>300
**Standard**	Ascorbic acid	6.69 ± 0.02

>300 μg/mL: Inactive, <10 μg/mL: highly active, 50 to 150 μg/mL: moderately active, 150 to 300 μg/mL: weakly active.

### Antimicrobial activity

The results of the agar diffusion assay indicated that most of the crude extracts did not show significant inhibition activity towards targeted microbes. Some crude extracts of different parts of the plant displayed either weak or moderate activities with inhibition zones between 6 and 13 mm.

- In this study, only the isolated compound methyl gallate (1) demonstrated strong antibacterial activity towards *MRSA* with an inhibition zone of 21.5 mm, as compared with an inhibition zone of 23.0 mm given by the standard compound (streptomycin). In addition, methyl gallate (1) also exhibited moderate antimicrobial activities towards *P. aeruginosa*, *S. choleraesuis* and *B. subtilis* with inhibition zones 12.0, 15.5, and 12.5 mm, respectively. This was in agreement with previous reports on the potent antimicrobial properties of methyl gallate (1) [27] which showed high MIC (Minimum Inhibitory Concentration) values (MICs 17.5 - 48.3 μg/mL) towards *B. subtilis*; *Streptomyces viridochromogenes*; *S. aureus*; *Escherichia coli*; *Mucor miehei*; *Candida albicans*. Meanwhile, there were no inhibition zones shown by quercitrin (12), mangiferonic acid (4), 3*β*-hydroxy-cycloart-24-ene-26-oic (5) and lupeol (7) towards the targeted microbes.
- As for the antifungal tests, none of extracts and isolated compounds showed activity against *Candida albican*, *Aspergillus ochraceaus* and *Sacchoromyces cerevisiae*. To our knowledge, this is the first antimicrobial activity study reported on *Mangifera pajang*.

## Conclusions

Extracts of the kernel, stem bark and leaves of *Mangifera pajang* have demonstrated potential cytotoxic activity towards MCF-7, HeLa and HT-29 cancer cells, and the extract of kernel also displayed strong free radical scavenging activity. These are assumed to be due to the presence of bioactive constituents that were isolated including cycloartane and lupane triterpenes, together with the major constituent, methyl gallate. The plant species, *Mangifera pajang* could become a potential source for natural anticancer (especially for breast, liver and colon cancers) and antioxidant agents. In particular, the major isolated constituent, methyl gallate could become a potential anticancer, antioxidant and antimicrobial agent.
